# Molecular study of sapovirus in acute gastroenteritis in children: a cross-sectional study

**DOI:** 10.12688/f1000research.29991.1

**Published:** 2021-02-17

**Authors:** Maysaa El Sayed Zaki, Raghdaa Shrief, Rasha H. Hassan

**Affiliations:** 1 Clinical Pathology Department, Faculty of Medicine, Mansoura University, Mansoura, 35516, Egypt; 2 Medical Microbioogy and Immunology Department, Faculty of Medicine, Damietta University, Mansoura, 35516, Egypt; 3 Pediatrics Department, Faculty of Medicine, Mansoura University, Mansoura, 35516, Egypt

**Keywords:** sapovirus

## Abstract

**Background:** Sapovirus has emerged as a viral cause of acute gastroenteritis. However, there are insufficient data about the presence of this virus among children with acute gastroenteritis. The present study aimed to evaluate the presence of sapovirus in children with acute gastroenteritis by reverse transcriptase-polymerase chain reaction (RT-PCR).

**Methods:** A cross-sectional study enrolled 100 children patients with acute gastroenteritis from outpatient clinics with excluded bacterial pathogens and parasitic infestation. A stool sample was collected from each child for laboratory examination. Each stool sample was subjected to study by direct microscopic examination, study for rotavirus by enzyme-linked immunoassay (ELISA) and the remaining sample was subjected to RNA extraction and RT- PCR for sapovirus.

**Results:** The most frequently detected virus was rotavirus by ELISA (25%). RT-PCR detected sapovirus in 7% of the stool samples. The children with sapovirus were all from rural regions and presented mainly during the winter season in Egypt (42.9%). The main presenting symptoms were fever (71.4%) and vomiting (57.1%). None of the children with sapovirus had dehydration. Rotavirus was significantly associated with sapovirus infections in 5 patients (71.4%, P=0.01). There was an insignificant difference between symptoms of gastroenteritis in children with sapovirus and children with gastroenteritis without sapovirus as regards vomiting (P=0.7), fever (P=0.46), and abdominal pain (P=0.69).

**Conclusion:** The present study highlights the emergence of sapovirus as a frequent pathogen associated with acute gastroenteritis in children. There is a need for a national survey program for the study of sapovirus among other pathogens associated with acute gastroenteritis for better management of such infection.

## Introduction

Sapovirus is a single strand non enveloped RNA virus that belongs to the Caliciviridae family ^1– 4^. The length of its genome is 7.1 to 7.7 kb, and its polyadenylated 3’ terminal is responsible for viral replication, while its 5’ terminal is associated with viral translation through production of VPg ^5, 6^. The viral genome consists of two to three open reading frames ^5^. There are 15 genotypes of the virus with only four genotypes that can infect humans, namely GI, GII, GIV and GV) ^2, 5, 7, 8^.

The laboratory methods for detection of sapovirus include enzyme-linked immunosorbent assay, electron microscopy, reverse transcription-polymerase chain reaction (RT-PCR) and next-generation sequencing ^3^. The sensitivity and specificity of RT-PCR are good and can be used for routine identification of the virus ^3^. The primers used for the amplification of the virus depend upon the use of a segment from VP1 encoding gene compared to the RdRp region ^3, 9^. The classification of sapovirus depends upon complete sequence analysis of VP1 ^6, 10, 11^. 

Sapovirus infection is associated with viral gastroenteritis, especially in children below five with, around two million deaths around the world, and sapovirus causing viral gastroenteritis represents the second common cause of death ^12^. The common symptoms are vomiting and diarrhoea ^5^. The transmission of sapovirus occurs via ingestion of contaminated food and water and also by direct contact with affected individuals ^13, 14^. The infection occurs both sporadically and as an outbreak ^2, 9^. Treatment is symptomatic to prevent the aggravation of the disease ^3, 9^, and prevention of the infection depends mainly upon access to clean drinking water and food ^15^.

There are data available about sapovirus infection in different countries such as Peru ^16^ Iran ^17^ and Ethiopia ^18^. However, to our best of knowledge, there are no available reports about this in Egypt. Therefore, the present study aimed to evaluate the presence of sapovirus in children with acute gastroenteritis by RT-PCR.

## Methods

### Study design and participants

The present study was a cross-sectional study that enrolled 100 children patients (sample size calculated by a statistician) with acute gastroenteritis from outpatient clinics from Mansoura Children’s Hospital, Mansoura, Egypt within the previous 48 hours with excluded bacterial pathogens and parasitic infestation by microbiological culture and microscopic examination to exclude parasites. The study was carried out from January 2019 till February 2020. 

The procedures followed were approved by the Mansoura Faculty of Medicine, Egypt ethical committee on human experimentation (R.20.11.1053) and were carried out in accordance with the Helsinki Declaration of 1975, as revised in 1983. Written informed consent was obtained from the parents of the included children.

Each child was subjected to full medical history by asking the parents about the residence area and age of the child, which was then followed by clinical examination. A stool sample was collected from each child for laboratory examination. 

### Stool samples

Each stool sample was subjected to study by direct microscopic examination, study for rotavirus by ELISA Ridascreen ^®^ (R-Biopharm AG- An der Neuen Bergstraße 1764297 Darmstadt, Germany), and the remaining samples was subjected to RNA extraction and RT-PVR for sapovirus.

***ELISA for rotavirus.*** The kit is ELISA for qualitative detection for rotavirus in the stool sample. The kit used monoclonal antibodies in a sandwich-type method. The monoclonal antibody is directed to the protein of the six viral genes (VP6), and it is used for the coating of the wells of the microplate. The VP6 is a group-specific antigen present in all rotaviruses genotypes. In brief, the stool suspension was prepared using ready to use stool diluent (protein-buffered NaCl solution). A total of 1 ml of the diluent was added to 100 microliter of the stool and homogenized the suspension by aspiration into and ejection from a disposable pipette or, alternatively, blending in a Vortex mixer. The suspension was allowed to stand for a short period of time (10 minutes) for the coarse stool particles to settle, and the clarified supernatant of the stool suspension was used directly in the test.

The control supplied with the kit were added to the wells of a microplate with biotinylated monoclonal anti-rotavirus antibodies and incubated at room temperature for one hour. The wells were washed with the supplied wash buffer after incubation and streptavidin poly-peroxidase conjugate was added before a second incubation was performed for a half-hour at room temperature. After a second wash, the substrate of the enzyme was added leading to a colour change of colourless to blue if the test is positive. The samples were then incubated at room temperature for 15 minutes. A stop reagent was added, changing the colour from blue to yellow. The colour intensity was proportional to the concentration of rotavirus present in the stool samples in comparison with a control, and was measured at 450 nm using a microplate ELISA reader (Statfax Chromate 4300). 

### Sapovirus PCR

***Extraction of RNA and complementary DNA preparation.*** The stool samples were subjected to the extraction of RNA of sapovirus using QIAamp Viral RNA kit (Qiagen). The extraction was performed according to the instructions supplied by the manufacturer.

Complementary DNA (cDNA) was generated by adding 100 nanograms of the extracted RNA to 32.5 µl prepared mixture composed of 1 µl hexamers primers (Fermentas, Latvia), 4 µl of 5 × buffer, 0.5 µl of Ribolock RNase inhibitor, 2 µl of dNTP mixture composed of 10 mM each of dNTP, 200 units of reverse transcriptase enzyme and 19 µ1 of RNase free water (Fermentas, Latvia). The RT assay was performed at 42°C for one hour.

***PCR for sapovirus.*** The amplification process was carried out using previously reported primers with nucleotide sequences of primers as follows: SLV5317 forward 5’-CGGRCYTCAAAVSTACCBCCCCA-3’; SLV5317 reverse 5’- CTCGCCACCTACRAWGCBTGGTT-3’ ^19^.

The amplification mixture used was ready to use Qiagen *Taq* PCR Master Mix Kit mixture (Cat No./ID: 201443) with 5 µl of cDNA added to a 20 µl PCR mix. The amplification procedures were performed using the following conditions: denaturation at 94°C for 5 minutes, then 35 cycles composed of 94°C for 45 seconds- 55°C for 45 seconds and 72°C for 1 minute, then final extension of 7 minutes at 72°C (MiniAmp Thermal Cycler, Applied Biosystem).

. PCR products were visualised under UV illumination after electrophoresis on a 1% agarose gel stained with ethidium bromide. The estimated amplified fragment size for sapovirus was 434 bp ^19^.

### Statistical analysis

Data were analysed with SPSS 22 (SPSS Inc, Chicago, Illinois, USA). Qualitative values were calculated as numbers and percentages. The use of the chi-square test performed comparisons, and the P-value was considered significant if it was <0.05.

## Results

The study included 100 children with acute gastroenteritis manifested by diarrhoea associated predominately with fever (56%), vomiting (47%), abdominal pain (42%). A minority had dehydration (11%). Their mean age± SD was 53.33± 11.71 months. Most cases presented in the spring season (34%) followed by winter (24%). Demographics of the children are shown in Table 1.

**Table 1.** Demographic and clinical data of the studied children (n=100).

Parameter	
Age, months (mean± SD)	53.33± 11.71
Gender, n (%) *Male* *Female*	66 (66) 34 (34)
Season, n (%) *Summer (21 June-22 September)* *Autumn (22 September-21 December)* *Winter (22 September -20 March)* *Spring (20 March-21 June)*	20 (20) 22 (22) 24 (24) 34 (34)
Residence, n (%) *Rural* *Urban*	68 (68) 32 (32)
Abdominal pain, n (%)	42 (42)
Fever, n (%)	56 (56)
Vomiting, n (%)	47 (47)
Dehydration, n (%)	11 (11)

The most frequently detected virus was rotavirus by ELISA (25%). RT-PCR detected sapovirus in 7% of the stool samples.

The children with sapovirus were all from rural regions (Belkas, Dekrnes, Aga) and presented mainly during the winter season in Egypt (42.9%). The main presenting symptoms were fever (71.4%) and vomiting (57.1%). None of the children with sapovirus had dehydration. Rotavirus was significantly associated with sapovirus infections in 5 patients (71.4%, P=0.01). There was an insignificant difference between symptoms of gastroenteritis in children with sapovirus and children with gastroenteritis without sapovirus as regards vomiting (P=0.7), fever (P=0.46), abdominal pain (P=0.69) ( Table 2).

**Table 2.** Comparison between sapovirus positive children and sapovirus negative children.

	Sapovirus positive (n=7)	Sapovirus negative (n=93)	Odds ratio	95%CI	P-value
	n (%)				
Gender *Male* *Female*	4 (57.1) 3 (42.9)	62 (66.7) 31 (33.3)	0.667	0.14-3.155	0.61
Age, months (mean± SD)	48.86± 9.40	51.52± 11.88			0.6 F=0.33
Rural residence	7 (100)	61 (65.6)	0.897	0.828-0.972	0.09
Season *Summer (21 June-22 Sept)* *Autumn (22 Sept-21 December)* *Winter (22 September -20 March)* *Spring (20 March-21 June)*	0 (0) 2 (28.6) 3 (42.9) 2 (28.6)	20 (21.5) 20 (21.5) 21 (22.6) 32 (34.4)	1.55	0.32-7.315	0.41
Vomiting	4 (57.1)	43 (46.2)	1.6	0.32-7.31	0.7
Abdominal pain	2 (28.6)	40 (43.01)	0.53	0.1-2.87	0.69
Fever	5 (71.4)	51 (54.8)	2.059	0.38-11.157	0.46
Dehydration	0 (0)	11 (11.8)	1.085	1.02-1.15	0.43
Rotavirus	5 (71.4)	20 (21.5)	9.12	1.646-50.594	0.01

## Discussion

Viral pathogens represent a significant aetiology for acute gastroenteritis. These infections are usually self-limited in developed countries while it may lead to mortality in underdeveloped countries, especially in children ^13^.

The present study included 100 children with acute gastroenteritis manifested by diarrhoea with the majority experiencing fever (56%), vomiting (47%), and abdominal pain (42%). A minority had dehydration (11%). These are common symptoms of viral gastroenteritis. These patients usually present to outpatient clinics and do not require hospital admission except for dehydration ^20^. 

The proper management of children with acute gastroenteritis relies upon appropriate and robust diagnosis of the aetiology. In the present study, the most frequently detected virus was rotavirus by ELISA (25%). Previous meta-analysis study revealed that rotavirus is associated with acute gastroenteritis in 31.5% of children and 25.7% in the general population ^14^. Previous studies in Africa reported that the prevalence of rotavirus infections ranged from 22.73% up to 30% ^21, 22^. A previous study from Egypt reported that the prevalence of rotavirus was 31% among children with acute gastroenteritis ^23^.

The study of sapovirus as an emerging pathogen associated with acute gastroenteritis has gained importance in recent years. Research has been facilitated by the emergence of the molecular techniques in laboratory diagnosis ^16, 24, 25^. In the present study, sapovirus was detected among 7% of children with acute gastroenteritis by RT-PCR. A previous meta-analysis study reported that the prevalence of sapovirus was 6.5% with a remarkable difference in the presence of sapovirus between low income and high-income countries ^26^. Another study reported a lower prevalence of sapovirus 4.6% (10/219) ^27^. Previous studies reported the prevalence of 3% up to 17% of sapovirus in acute gastroenteritis in children with gastroenteritis in high and low-income countries, respectively ^16, 24, 25^. The variation of the prevalence rates reported may be due to the variation of the climate, environment, socio-economic factors, and cultural practices beside the difference of the used method of diagnoses.

The management of sapovirus depends mainly upon oral rehydration solution and zinc supplementation ^28^. The risk factors for sapovirus infection are not fully understood. The prevention of sapovirus infection depends mainly upon efficient hand hygiene practice, environmental disinfection, proper sewage disposal, and limited contact with ill individuals. There is an argument about the role of improvement of water sanitation in the prevention of sapovirus as it is a common pathogen in both high and low-income countries. However, as it is transmitted by contaminated water and food ^29^, increasing food and water sanitation will reduce the burden of the infection.

The use of new molecular technologies for sapovirus detection in different samples from patients, food and environment, is mandatory to recognize the mode of sapovirus transmission. Infection at a young age may predispose to durable immunity. Therefore, the development of a vaccine toward this virus may reduce the burden of this infection ^30^.

In the present study, there was an insignificant difference between the clinical presentation of sapovirus positive and sapovirus negative children. The clinical symptoms associated with acute gastroenteritis usually include diarrhoea, vomiting, and fever, making laboratory diagnosis essential for appropriate management. Therefore, there is a need for a national survey program to improve the monitoring of the circulation of the new virus such as sapovirus alongside other pathogens associated with gastroenteritis to improve the control measures ^30^.

## Conclusions

The present study highlights the emergence of sapovirus as a frequent pathogen associated with acute gastroenteritis in children. There is a need for a national survey program for the study of sapovirus among other pathogens association with acute gastroenteritis for better management of such infection.

## Data availability

### Underlying data

Figshare: Molecular study of sapovirus in acute gastroenteritis in children: a cross-sectional study, https://doi.org/10.6084/m9.figshare.13574933.v1 ^31^

Data are available under the terms of the Creative Commons Attribution 4.0 International license (CC-BY 4.0).

## References

[1] Lauritsen KT Hansen MS Johnsen CK : Repeated examination of natural sapovirus infections in pig litters raised under experimental conditions. *Acta Vet Scand.* 2015;57:60. 10.1186/s13028-015-0146-7 26410386 PMC4583762

[2] Liu X Jahuira H Gilman RH : Etiological Role and Repeated Infections of Sapovirus among Children Aged Less than 2 Years in a Cohort Study in a Peri-urban Community of Peru. *J Clin Microbiol.* 2016;54(6):1598–1604. 10.1128/JCM.03133-15 27076657 PMC4879303

[3] Oka T Wang Q Katayama K : Comprehensive review of human sapoviruses. *Clin Microbiol Rev.* 2015;28(1):32–53. 10.1128/CMR.00011-14 25567221 PMC4284302

[4] Makhaola K Moyo S Kebaabetswe LP : Distribution and Genetic Variability of Sapoviruses in Africa. *Viruses.* 2020;12(5):490. 10.3390/v12050490 32349380 PMC7291139

[5] Li J Shen Q Zhang W : Genomic organization and recombination analysis of a porcine sapovirus identified from a piglet with diarrhea in China. *Virol J.* 2017;14(1):57. 10.1186/s12985-017-0729-1 28302145 PMC5356244

[6] Vinjé J Estes MK Esteves P : ICTV Virus Taxonomy Profile: *Caliciviridae.* *J Gen Virol.* 2019;100(11):1469–1470. 10.1099/jgv.0.001332 31573467 PMC7011698

[7] Oka T Lu Z Phan T : Genetic Characterization and Classification of Human and Animal Sapoviruses. *PLoS One.* 2016;11(5):e0156373. 10.1371/journal.pone.0156373 27228126 PMC4881899

[8] Varela MF Rivadulla E Lema A : Human Sapovirus among Outpatients with Acute Gastroenteritis in Spain: A One-Year Study. *Viruses.* 2019;11(2):144. 10.3390/v11020144 30744057 PMC6409837

[9] Liu X Yamamoto D Saito M : Molecular detection and characterization of sapovirus in hospitalized children with acute gastroenteritis in the Philippines. *J Clin Virol.* 2015;68:83–88. 10.1016/j.jcv.2015.05.001 26071343

[10] Hansman GS Oka T Sakon N : Antigenic diversity of human sapoviruses. *Emerg Infect Dis.* 2007;13(10):1519–1525. 10.3201/eid1310.070402 18258001 PMC2851512

[11] Harada S Tokuoka E Kiyota N : Phylogenetic analysis of the nonstructural and structural protein encoding region sequences, indicating successive appearance of genomically diverse sapovirus strains from gastroenteritis patients. *Jpn J Infect Dis.* 2013;66(5):454–457. 10.7883/yoken.66.454 24047751

[12] Kebede Fufa W Berhe Gebremedhin G Gebregergs GB : Assessment of Poor Home Management Practice of Diarrhea and Associated Factors among Caregivers of Under-Five Years Children in Urban and Rural Residents of Doba Woreda, Ethiopia: Comparative Cross-Sectional Study. *Int J Pediatr.* 2019;2019:8345245. 10.1155/2019/8345245 31275402 PMC6582835

[13] Shane AL Mody RK Crump JA : 2017 Infectious Diseases Society of America Clinical Practice Guidelines for the Diagnosis and Management of Infectious Diarrhea. *Clin Infect Dis.* 2017;65(12):e45–e80. 10.1093/cid/cix669 29053792 PMC5850553

[14] Ojobor CD Olovo CV Onah LO : Prevalence and associated factors to rotavirus infection in children less than 5 years in Enugu State, Nigeria. *VirusDis.* 2020;31(3):1–7. 10.1007/s13337-020-00614-x 32837972 PMC7409042

[15] Varela MF Ouardani I Kato T : Sapovirus in Wastewater Treatment Plants in Tunisia: Prevalence, Removal, and Genetic Characterization. *Appl Environ Microbiol.* 2018;84(6):e02093–17. 10.1128/AEM.02093-17 29305515 PMC5835752

[16] Sánchez GJ Mayta H Pajuelo MJ : Epidemiology of Sapovirus Infections in a Birth Cohort in Peru. *Clin Infect Dis.* 2018;66(12):1858–1863. 10.1093/cid/cix1103 29309577 PMC5982808

[17] Romani S Azimzadeh P Mohebbi SR : Prevalence of sapovirus infection among infant and adult patients with acute gastroenteritis in Tehran, Iran. *Gastroenterol Hepatol Bed Bench.* 2012;5(1):43–8. 24834197 PMC4017447

[18] Gelaw A Pietsch C Mann P : Molecular detection and characterisation of sapoviruses and noroviruses in outpatient children with diarrhoea in Northwest Ethiopia. *Epidemiol Infect.* 2019;147:e218. 10.1017/S0950268819001031 31364546 PMC6625200

[19] Svraka S Vennema H van der Veer B : Epidemiology and genotype analysis of emerging sapovirus-associated infections across Europe. *J Clin Microbiol.* 2010;48(6):2191–8. 10.1128/JCM.02427-09 20392905 PMC2884469

[20] Stuempfig ND Seroy J : Viral Gastroenteritis. In: *StatPearls*. Treasure Island (FL): StatPearls Publishing;2020[Updated 2020 Jun 25]. Reference Source

[21] Djeneba O Damintoti K Denise I : Prevalence of rotavirus, adenovirus and enteric parasites among pediatric patients attending Saint Camille Medical Centre in Ouagadougou. *Pak J Biol Sci.* 2007;10(23):4266–70. 10.3923/pjbs.2007.4266.4270 19086583

[22] Mwenda JM Ntoto KM Abebe A : Burden and epidemiology of rotavirus diarrhea in selected African countries: preliminary results from the African Rotavirus Surveillance Network. *J Infect Dis.* 2010;202 Suppl:S5–11. 10.1086/653557 20684718

[23] Kamal Allayeh A Mostafa El-Baz R Mohamed Saeed N : Detection and Genotyping of Viral Gastroenteritis in Hospitalised Children Below Five Years Old in Cairo, Egypt. *Arch Pediatr Infect Dis.* 2018;6(3):e60288. 10.5812/pedinfect.60288

[24] Diez-Valcarce M Castro CJ Marine RL : Genetic diversity of human sapovirus across the Americas. *J Clin Virol.* 2018;104:65–72. 10.1016/j.jcv.2018.05.003 29753103 PMC8972816

[25] Hassan F Kanwar N Harrison CJ : Viral Etiology of Acute Gastroenteritis in <2-Year-Old US Children in the Post-Rotavirus Vaccine Era. *J Pediatric Infect Dis Soc.* 2019;8(5):414–421. 10.1093/jpids/piy077 30184153

[26] Magwalivha M Kabue JP Traore AN : Prevalence of Human Sapovirus in Low and Middle Income Countries. *Adv Virol.* 2018;2018:5986549, 12 pages. 10.1155/2018/5986549 30245718 PMC6139206

[27] Portal TM Reymão TKA Quinderé Neto GA : Detection and genotyping of enteric viruses in hospitalized children with acute gastroenteritis in Belém, Brazil: Occurrence of adenovirus viremia by species F, types 40/41. *J Med Virol.* 2019;91(3):378–384. 10.1002/jmv.25321 30231194

[28] Kobayashi S Fujiwara N Yasui Y : A foodborne outbreak of sapovirus linked to catered box lunches in Japan. *Arch Virol.* 2012;157(10):1995–1997. 10.1007/s00705-012-1394-8 22752792

[29] Rockx B De Wit M Vennema H : Natural history of human *calicivirus* infection: a prospective cohort study. *Clin Infect Dis.* 2002;35(3):246–53. 10.1086/341408 12115089

[30] Becker-Dreps S Bucardo F Vinjé J : Sapovirus: an important cause of acute gastroenteritis in children. *Lancet Child Adolesc Health.* 2019;3(11):758–759. 10.1016/S2352-4642(19)30270-6 31439497 PMC7213765

[31] Zaki M Shrief R Hassan R : Molecular study of sapovirus in acute gastroenteritis in children: a cross-sectional study. *figshare.* Dataset.2021. 10.6084/m9.figshare.13574933.v1

