# Molecular Detection of Sapovirus in Children Under Five Years with Acute Gastroenteritis in Mansoura, Egypt between January 2019 and February 2020

**DOI:** 10.12688/f1000research.29991.4

**Published:** 2021-11-24

**Authors:** Maysaa El Sayed Zaki, Raghdaa Shrief, Rasha H. Hassan

**Affiliations:** 1 Clinical Pathology Department, Faculty of Medicine, Mansoura University, Mansoura, 35516, Egypt; 2 Medical Microbioogy and Immunology Department, Faculty of Medicine, Damietta University, New Damietta, 34511, Egypt; 3 Pediatrics Department, Faculty of Medicine, Mansoura University, Mansoura, 35516, Egypt

**Keywords:** sapovirus

## Abstract

**Background: **Sapovirus has emerged as a viral cause of acute gastroenteritis. However, there is limited data on sapovirus in Egypt. . The present study aimed to evaluate the presence of sapovirus in children with acute gastroenteritis <5 years in Mansoura, Egypt from January 2019 to February 2020 by reverse transcriptase-polymerase chain reaction (RT-PCR).

**Methods: **The cross-sectional study enrolled a 100 children <5 years who presented with acute gastroenteritis at an outpatient clinic in Mansoura, Egypt between January 2019 and February 2020. Clinical data, demographic data and a stool sample was collected from each child. Stools were screened by microscopy for parasites and culture methods for bacteria and excluded from the study if positive for either. Specimens were also screened for rotavirus by enzyme immune assays (EIA) and sapovirus by reverse transcription PCR.

**Results: **The most frequently detected virus was rotavirus by ELISA 25% (25/100). RT-PCR detected sapovirus in 7% (7/100) of the stool samples. The children with sapovirus were all from rural regions and presented mainly during the winter season in Egypt 42.9% (3/7). The main presenting symptoms were fever 71.4% (5/7) and vomiting 57.1% (4/7). None of the children with sapovirus had dehydration. Rotavirus was significantly associated with sapovirus infections in  five samples (5/7) , 71.4%, P=0.01.

**Conclusion: **The present study highlights the emergence of sapovirus as a frequent pathogen associated with acute gastroenteritis in children. There is a need for a national survey program for the study of sapovirus among other pathogens associated with acute gastroenteritis for better management of such infection.

## Introduction

Sapovirus is a single strand non enveloped RNA virus that belongs to the *Caliciviridae* family ^1– 4^. The length of its genome is 7.1 to 7.7 kb, and its polyadenylated 3’ terminal is responsible for viral replication, while its 5’ terminal is associated with viral translation through production of VPg ^5, 6^. The viral genome consists of two to three open reading frames ^5^. There are 15 genogroups of the virus with only four of them can infect humans, namely GI, GII, GIV and GV) ^2, 5, 7, 8^.

The laboratory methods for detection of sapovirus include enzyme-linked immunosorbent assay, electron microscopy, reverse transcription-polymerase chain reaction (RT-PCR) and next-generation sequencing ^3^. The sensitivity and specificity of RT-PCR are good and can be used for routine identification of the virus ^3^. The sensitivity and specificity of most RT-PCR detection assays for sapovirus are above 90% ^9, 10^. The primers used for the amplification of the virus depend upon the use of a segment from capsid region ^3, 11^. The classification of sapovirus depends upon complete sequence analysis of VP1 ^6, 12, 13^.

Diarrhea represents the second common cause of death in children and is associated with about 525 000 deaths around the world ^14^. Sapovirus gastroenteritis occurs via ingestion of contaminated food and water and also by direct contact with affected individuals ^13– 16^. The infection occurs both sporadically and as an outbreak ^2, 11^. Treatment is symptomatic to prevent the aggravation of the disease ^3, 11^, and prevention of the infection depends mainly upon access to clean drinking water and food and on good hygiene habits (WHO’s five keys to food safety) and hand washing ^17^.

There are data available about sapovirus infection in different countries such as Peru ^18^ Iran ^19^ and Ethiopia ^20^. There is limited data on sapovirus in Egypt. Therefore, the present study aimed to evaluate the presence of sapovirus in children with acute gastroenteritis by RT-PCR.

## Methods

### Study design and participants

The cross-sectional study enrolled 100 children <5 years who presented with acute gastroenteritis at an outpatient clinic in Mansoura, Egypt between January 2019 and February 2020. Clinical data, demographic data and a stool sample was collected from each child. Stools were screened by microscopy for parasites and culture methods for bacteria and excluded from the study if positive for either. Specimens were also screened for rotavirus by enzyme immune assays (EIA) and sapovirus by reverse transcription PCR.

The procedures followed were approved by the Mansoura Faculty of Medicine, Egypt ethical committee on human experimentation (R.20.11.1053) and were carried out in accordance with the Helsinki Declaration of 1975, as revised in 1983. Written informed consent was obtained from the parents of the included children.

Each child was subjected to full medical history by asking the parents about the residence area and age of the child, which was then followed by clinical examination. A stool sample was collected from each child for laboratory examination.

### Stool samples

Each stool sample was subjected to study by direct microscopic examination, study for rotavirus by ELISA Ridascreen ^®^ (R-Biopharm AG- An der Neuen Bergstraße 1764297 Darmstadt, Germany), and the remaining samples was subjected to RNA extraction and RT-PCR for sapovirus.

***ELISA for rotavirus.*** The ELIA was completed according to the manufacturer’s instructions with EIA plates read at 450nm on a Statfax Chromate 4300 (Unit No. 518, 5th Floor, MGF Metropolis Mall, MG Road, Gurgaon, Haryana-122002).

### Sapovirus PCR

***Extraction of RNA and complementary DNA preparation.*** The stool samples were subjected to the extraction of RNA of sapovirus using QIAamp Viral RNA kit (Qiagen). The extraction was performed according to the instructions supplied by the manufacturer.

### Reverse transcriptase

A total volume of reaction mixture were prepared by adding 7.5 µl of extracted RNA to 2.05 µl 5× First-Strand Buffer (Invitrogen-USA), 0.75 µl of 10 mM dNTPs (Qiagen-USA), 0.375 µl (1 µg/ µl) of random primer (Qiagen-USA), 0.75 µl of 10 mM DTT (Invitrogen), 0.5 µl of RNase Inhibitor (Qiagen-USA), and 0.75 µl (200 U/ µl) of SuperScript Reverse Transcrip tase II (Thermofisher-USA). MilliQ water was added to give a total volume of 15.0 µl.

***PCR for sapovirus.*** The amplification process was carried out using previously reported primers with nucleotide sequences of primers as follows: SLV5749 forward 5’-CGGRCYTCAAAVSTACCBCCCCA-3’; SLV5317 reverse 5’- CTCGCCACCTACRAWGCBTGGTT-3’ ^9^.

The cDNA generated from the previous step was used as 2.5 µl and added to ready to use amplification mixture supplied from Qiagen with 0.4 µl of the used primers in total volume 25 µl. The amplification procedures were performed using the following conditions: denaturation at 94°C for 5 minutes, then 35 cycles composed of 94°C for 45 seconds- 55°C for 45 seconds and 72°C for 1 minute, then final extension of 7 minutes at 72°C (MiniAmp Thermal Cycler, Applied Biosystem).

PCR products were visualized under UV illumination after electrophoresis on a 1% agarose gel stained with ethidium bromide. The estimated amplified fragment size for sapovirus was 434 bp ^9^.

### Statistical analysis

Data were analysed with SPSS 22 (SPSS Inc, Chicago, Illinois, USA). Quantitative values were calculated as numbers and percentages. The use of the chi-square test performed comparisons, and the P-value was considered significant if it was <0.05.

## Results

The study included 100 children with AGE manifested by diarrhoea associated predominately with fever (56%) 56/100, vomiting (47%) 47/100, abdominal pain (42%) 42/100. A minority had dehydration (11%) 11/100. Their mean age± SD was 53.33± 11.71 months. Most cases presented in the spring season (34%) 34/100 followed by winter (24%) 24/100. Demographics of the children are shown in Table 1.

**Table 1.** Demographic and clinical data of the studied children (n=100).

Parameter	
Age, months (mean± SD)	53.33± 11.71
Gender, n (%) *Male* *Female*	66 (66) 34 (34)
Season, n (%) *Summer (21 Ęune-22 September)* *Autumn (23 September-21 December)* *Winter (22 December-19 March)* *Spring (20 March-20 Ęune)*	20 (20) 22 (22) 24 (24) 34 (34)
Residence, n (%) *Rural* *Urban*	68 (68) 32 (32)
Abdominal pain, n (%)	42 (42)
Fever, n (%)	56 (56)
Vomiting, n (%)	47 (47)
Dehydration, n (%)	11 (11)

The most frequently detected virus was rotavirus by ELISA (25%) 25/100. RT-PCR detected sapovirus in seven samples 7% 7/100 of the stool samples.

The children with sapovirus were all from rural regions (Belkas, Dekrnes, Aga) and presented mainly during the winter season (22 December–19 March) in Egypt in three children (42.9%) 3/7. The main presenting symptoms were fever in five children (71.4%) 5/7 and vomiting in four children (57.1%) 4/7. None of the children with sapovirus had dehydration. Rotavirus was significantly associated with sapovirus infections in five children (71.4%, P=0.01) 5/7 ( Table 2).

**Table 2.** Comparison between sapovirus positive children and sapovirus negative children.

	Sapovirus positive (n=7)	Sapovirus negative (n=93)	Odds ratio	95%CI	P-value
	n (%)				
Gender *Male* *Female*	4 (57.1) 3 (42.9)	62 (66.7) 31 (33.3)	0.667	0.14-3.155	0.61
Age, months (mean± SD)	48.86± 9.40	51.52± 11.88			0.6 F=0.33
Rural residence	7 (100)	61 (65.6)	0.897	0.828-0.972	0.09
Season *Summer (21 Ęune-22 September)* *Autumn (23 September-21 December)* *Winter (22 December-19 March)* *Spring (20 March-20 Ęune)*	0 (0) 2 (28.6) 3 (42.9) 2 (28.6)	20 (21.5) 20 (21.5) 21 (22.6) 32 (34.4)	1.55	0.32-7.315	0.41
Vomiting	4 (57.1)	43 (46.2)	1.6	0.32-7.31	0.7
Abdominal pain	2 (28.6)	40 (43.01)	0.53	0.1-2.87	0.69
Fever	5 (71.4)	51 (54.8)	2.059	0.38-11.157	0.46
Dehydration	0 (0)	11 (11.8)	1.085	1.02-1.15	0.43
Rotavirus	5 (71.4)	20 (21.5)	9.12	1.646-50.594	0.01

## Discussion

Viral pathogens represent a significant aetiology for acute gastroenteritis. These infections are usually self-limited in high income countries while it may lead to mortality in low income countries, especially in children ^15, 21^.

The present study including children <5 with diarrhea in an outpatient setting showed that the majority of cases experienced fever, vomiting and abdominal pain; common symptoms of viral gastroenteritis. These patients usually present to outpatient clinics and do not require hospital admission except in rare instance of dehydration. The findings support the results noted in previous study ^22^.

The proper management of children with AGE relies upon appropriate and robust diagnosis of the aetiology. In the present study, the most frequently detected virus was rotavirus by ELISA (25%). A previous systematic review in African countries revealed that rotavirus is associated with AGE in 31.5% of children and 25.7% in the general population ^16^. Previous studies in Africa reported that the prevalence of rotavirus infections ranged from 22.73% up to 30% in children below 5 years ^23, 24^. The study of rotavirus in Africa was carried out from 2006 to 2008 in 11 African countries and 2200 samples out of 5461 stool was positive for rotavirus ^24^. A previous study pubkished 2018 from Abu El-reesh hospital in Cairo, Egypt reported that the prevalence of rotavirus was 31% among 119 hospitalized children below 5 years with AGE ^25^.

The study of sapovirus as an emerging pathogen associated with AGE has gained importance in recent years. Research has been facilitated by the emergence of the molecular techniques in laboratory diagnosis ^18, 26, 27^. In the present study, sapovirus was detected among 7% of children with AGE by RT-PCR. A previous meta-analysis study reported that the prevalence of sapovirus was 6.5% with a remarkable difference in the presence of sapovirus between low income and high-income countries ^28^. Another study reported a lower prevalence of sapovirus 4.6% (10/219) ^28^. The incidence of sapovirus infection in a study from Puru published in 2018 in the first and second years of life was 4.3 and 11.1 per 100 child-months, respectively ^18^. In a case-control study from United states of America published 2019 in 300 children below 2 years with AGE versus 272 matched healthy control the prevalence of sapovirus was 7.0% versus 3.0% (P = .07) ^29^. The variation of the prevalence rates reported may be due to the variation of the climate, environment, socio-economic factors, and cultural practices beside the difference of the used method of diagnoses.

The treatment of sapovirus depends mainly upon oral rehydration solution and zinc supplementation ^30^. The risk factors for sapovirus infection are not fully understood. The prevention of sapovirus infection depends mainly upon efficient hand hygiene practice, environmental disinfection, proper sewage disposal, and limited contact with ill individuals. There are conflicting data about the role of improvement of water sanitation in the prevention of sapovirus as it is a common pathogen in both high and low-income countries. However, as it is transmitted by contaminated water and food ^31^, improving food safety and access to clean water and improved sanitation services will reduce the burden of the infection.

The use of new molecular technologies for sapovirus detection in different samples from patients, food and environment, is important to recognize the mode of sapovirus transmission. Infection at a young age may predispose to durable immunity. Therefore, the development of a vaccine toward this virus may reduce the burden of this infection ^32^.

In the present study, there was no significant difference between the clinical presentation of sapovirus positive and sapovirus negative children. The clinical symptoms associated with AGE usually include diarrhoea, vomiting, and fever, making laboratory diagnosis essential for appropriate management. Therefore, there is a need for a national survey program to improve the monitoring of the circulation of enteric viruses including sapovirus alongside other pathogens associated with gastroenteritis to improve the control measures ^32^.

## Conclusions

The present study highlights the presence of sapovirus as a pathogen associated with AGE in children from Mansoura, Egypt during 2019 and 2020. There is a need for a national survey program for the study of sapovirus among other pathogens association with AGE for better management of such infection.

## Data availability

### Underlying data

Figshare: Molecular study of sapovirus in acute gastroenteritis in children: a cross-sectional study, https://doi.org/10.6084/m9.figshare.13574933.v1 ^33^

Data are available under the terms of the Creative Commons Attribution 4.0 International license (CC-BY 4.0).

## References

[1] Lauritsen KT Hansen MS Johnsen CK : Repeated examination of natural sapovirus infections in pig litters raised under experimental conditions. *Acta Vet Scand.* 2015;57:60. 10.1186/s13028-015-0146-7 26410386 PMC4583762

[2] Liu X Jahuira H Gilman RH : Etiological Role and Repeated Infections of Sapovirus among Children Aged Less than 2 Years in a Cohort Study in a Peri-urban Community of Peru. *J Clin Microbiol.* 2016;54(6):1598–1604. 10.1128/JCM.03133-15 27076657 PMC4879303

[3] Oka T Wang Q Katayama K : Comprehensive review of human sapoviruses. *Clin Microbiol Rev.* 2015;28(1):32–53. 10.1128/CMR.00011-14 25567221 PMC4284302

[4] Makhaola K Moyo S Kebaabetswe LP : Distribution and Genetic Variability of Sapoviruses in Africa. *Viruses.* 2020;12(5):490. 10.3390/v12050490 32349380 PMC7291139

[5] Li J Shen Q Zhang W : Genomic organization and recombination analysis of a porcine sapovirus identified from a piglet with diarrhea in China. *Virol J.* 2017;14(1):57. 10.1186/s12985-017-0729-1 28302145 PMC5356244

[6] Vinjé J Estes MK Esteves P : ICTV Virus Taxonomy Profile: *Caliciviridae.* *J Gen Virol.* 2019;100(11):1469–1470. 10.1099/jgv.0.001332 31573467 PMC7011698

[7] Oka T Lu Z Phan T : Genetic Characterization and Classification of Human and Animal Sapoviruses. *PLoS One.* 2016;11(5):e0156373. 10.1371/journal.pone.0156373 27228126 PMC4881899

[8] Varela MF Rivadulla E Lema A : Human Sapovirus among Outpatients with Acute Gastroenteritis in Spain: A One-Year Study. *Viruses.* 2019;11(2):144. 10.3390/v11020144 30744057 PMC6409837

[9] Yan H Yagyu F Okitsu S : Detection of norovirus (GI, GII), Sapovirus and astrovirus in fecal samples using reverse transcription single-round multiplex PCR. *J Virol Methods.* 2003;114(1):37–44. 10.1016/j.jviromet.2003.08.009 14599677

[10] Svraka S van der Veer B Duizer E : Novel approach for detection of enteric viruses to enable syndrome surveillance of acute viral gastroenteritis. *J Clin Microbiol.* 2009;47(6):1674–1679. 10.1128/JCM.00307-09 19339472 PMC2691081

[11] Liu X Yamamoto D Saito M : Molecular detection and characterization of sapovirus in hospitalized children with acute gastroenteritis in the Philippines. *J Clin Virol.* 2015;68:83–88. 10.1016/j.jcv.2015.05.001 26071343

[12] Hansman GS Oka T Sakon N : Antigenic diversity of human sapoviruses. *Emerg Infect Dis.* 2007;13(10):1519–1525. 10.3201/eid1310.070402 18258001 PMC2851512

[13] Harada S Tokuoka E Kiyota N : Phylogenetic analysis of the nonstructural and structural protein encoding region sequences, indicating successive appearance of genomically diverse sapovirus strains from gastroenteritis patients. *Jpn J Infect Dis.* 2013;66(5):454–457. 10.7883/yoken.66.454 24047751

[14] https://www.who.int/news-room/fact-sheets/detail/diarrhoeal-disease.

[15] Shane AL Mody RK Crump JA : 2017 Infectious Diseases Society of America Clinical Practice Guidelines for the Diagnosis and Management of Infectious Diarrhea. *Clin Infect Dis.* 2017;65(12):e45–e80. 10.1093/cid/cix669 29053792 PMC5850553

[16] Ojobor CD Olovo CV Onah LO : Prevalence and associated factors to rotavirus infection in children less than 5 years in Enugu State, Nigeria. *VirusDis.* 2020;31(3):1–7. 10.1007/s13337-020-00614-x 32837972 PMC7409042

[17] Varela MF Ouardani I Kato T : Sapovirus in Wastewater Treatment Plants in Tunisia: Prevalence, Removal, and Genetic Characterization. *Appl Environ Microbiol.* 2018;84(6):e02093-17. 10.1128/AEM.02093-17 29305515 PMC5835752

[18] Sánchez GJ Mayta H Pajuelo MJ : Epidemiology of Sapovirus Infections in a Birth Cohort in Peru. *Clin Infect Dis.* 2018;66(12):1858–1863. 10.1093/cid/cix1103 29309577 PMC5982808

[19] Romani S Azimzadeh P Mohebbi SR : Prevalence of sapovirus infection among infant and adult patients with acute gastroenteritis in Tehran, Iran. *Gastroenterol Hepatol Bed Bench.* 2012;5(1):43–8. 24834197 PMC4017447

[20] Gelaw A Pietsch C Mann P : Molecular detection and characterisation of sapoviruses and noroviruses in outpatient children with diarrhoea in Northwest Ethiopia. *Epidemiol Infect.* 2019;147:e218. 10.1017/S0950268819001031 31364546 PMC6625200

[21] Colston JM Faruque ASG Hossain MJ : Associations between Household-Level Exposures and All-Cause Diarrhea and Pathogen-Specific Enteric Infections in Children Enrolled in Five Sentinel Surveillance Studies. *Int J Environ Res Public Health.* 2020;17(21):8078. 10.3390/ijerph17218078 33147841 PMC7663028

[22] Stuempfig ND Seroy J : Viral Gastroenteritis. In: *StatPearls*. Treasure Island (FL): StatPearls Publishing;2020[Updated 2020 Jun 25]. Reference Source

[23] Djeneba O Damintoti K Denise I : Prevalence of rotavirus, adenovirus and enteric parasites among pediatric patients attending Saint Camille Medical Centre in Ouagadougou. *Pak J Biol Sci.* 2007;10(23):4266–70. 10.3923/pjbs.2007.4266.4270 19086583

[24] Mwenda JM Ntoto KM Abebe A : Burden and epidemiology of rotavirus diarrhea in selected African countries: preliminary results from the African Rotavirus Surveillance Network. *J Infect Dis.* 2010;202 Suppl:S5–11. 10.1086/653557 20684718

[25] Kamal Allayeh A Mostafa El-Baz R Mohamed Saeed N : Detection and Genotyping of Viral Gastroenteritis in Hospitalised Children Below Five Years Old in Cairo, Egypt. *Arch Pediatr Infect Dis.* 2018;6(3):e60288. 10.5812/pedinfect.60288

[26] Diez-Valcarce M Castro CJ Marine RL : Genetic diversity of human sapovirus across the Americas. *J Clin Virol.* 2018;104:65–72. 10.1016/j.jcv.2018.05.003 29753103 PMC8972816

[27] Hassan F Kanwar N Harrison CJ : Viral Etiology of Acute Gastroenteritis in <2-Year-Old US Children in the Post-Rotavirus Vaccine Era. *J Pediatric Infect Dis Soc.* 2019;8(5):414–421. 10.1093/jpids/piy077 30184153

[28] Portal TM Reymão TKA Quinderé Neto GA : Detection and genotyping of enteric viruses in hospitalized children with acute gastroenteritis in Belém, Brazil: Occurrence of adenovirus viremia by species F, types 40/41. *J Med Virol.* 2019;91(3):378–384. 10.1002/jmv.25321 30231194

[29] Magwalivha M Kabue JP Traore AN : Prevalence of Human Sapovirus in Low and Middle Income Countries. *Adv Virol.* 2018;2018:5986549. 10.1155/2018/5986549 30245718 PMC6139206

[30] Kobayashi S Fujiwara N Yasui Y : A foodborne outbreak of sapovirus linked to catered box lunches in Japan. *Arch Virol.* 2012;157(10):1995–1997. 10.1007/s00705-012-1394-8 22752792

[31] Rockx B De Wit M Vennema H : Natural history of human *calicivirus* infection: a prospective cohort study. *Clin Infect Dis.* 2002;35(3):246–53. 10.1086/341408 12115089

[32] Becker-Dreps S Bucardo F Vinjé J : Sapovirus: an important cause of acute gastroenteritis in children. *Lancet Child Adolesc Health.* 2019;3(11):758–759. 10.1016/S2352-4642(19)30270-6 31439497 PMC7213765

[33] Zaki M Shrief R Hassan R : Molecular study of sapovirus in acute gastroenteritis in children: a cross-sectional study. *figshare.* Dataset.2021. 10.6084/m9.figshare.13574933.v1

