# Supplementary material for: Molecular Detection of Sapovirus in Children Under Five Years with Acute Gastroenteritis in Mansoura, Egypt between January 2019 and February 2020
Source: F1000Res. 2021 Nov 24;10:123. Originally published 2021 Feb 17. [Version 4] doi: 10.12688/f1000research.29991.4 (PMC8630562; doi:10.12688/f1000research.29991.4)
Supplement: Peer review history

# Peer review: Reviewer response for version 4

**DOI:** 10.5256/f1000research.79575.r101105

**Published:** 2021-11-29

**Authors:** Nicola A. Page

**Affiliations:** 1 Center for Enteric Diseases, National Institute for Communicable Diseases (NICD), Johannesburg, South Africa

All corrections were made.

Is the work clearly and accurately presented and does it cite the current literature?

Partly

If applicable, is the statistical analysis and its interpretation appropriate?

Partly

Are all the source data underlying the results available to ensure full reproducibility?

Yes

Is the study design appropriate and is the work technically sound?

Partly

Are the conclusions drawn adequately supported by the results?

Partly

Are sufficient details of methods and analysis provided to allow replication by others?

No

Reviewer Expertise:

Expertise includes epidemiology of enteric viruses

I confirm that I have read this submission and believe that I have an appropriate level of expertise to confirm that it is of an acceptable scientific standard.

# Peer review: Reviewer response for version 4

**DOI:** 10.5256/f1000research.79575.r101104

**Published:** 2021-11-25

**Authors:** Marta Diez Valcarce

**Affiliations:** 1 Division of Viral Diseases, Centers for Disease Control and Prevention, Atlanta, GA, USA

After the revisions I approve the article for indexing.

Is the work clearly and accurately presented and does it cite the current literature?

Yes

If applicable, is the statistical analysis and its interpretation appropriate?

Yes

Are all the source data underlying the results available to ensure full reproducibility?

Yes

Is the study design appropriate and is the work technically sound?

Yes

Are the conclusions drawn adequately supported by the results?

Yes

Are sufficient details of methods and analysis provided to allow replication by others?

Yes

Reviewer Expertise:

Molecular epidemiology of gastroenteritis viruses

I confirm that I have read this submission and believe that I have an appropriate level of expertise to confirm that it is of an acceptable scientific standard.

# Peer review: Reviewer response for version 3

**DOI:** 10.5256/f1000research.77735.r99618

**Published:** 2021-11-16

**Authors:** Nicola A. Page

**Affiliations:** 1 Center for Enteric Diseases, National Institute for Communicable Diseases (NICD), Johannesburg, South Africa

The revised paper has been improved in the current version and describes the detection of sapovirus in children under five presenting to an outpatient clinic for the treatment of diarrhoea between January 2019 and February 2020 in Mansoura, Egypt. 

 The following minor errors were noted:

- Discussion, paragraph 1: Please rather use low-income, middle-income or high-income as these have been defined by the World Bank rather than underdeveloped which is not specific or well defined. This correction was noted in the previous review and has not been full corrected.
- Discussion, paragraph 2: Please delete the word hospital. The previous review indicated that either outpatient or hospital should be retained and the other word deleted as it was unclear where the study was conducted. 
- Discussion, paragraph 3, line 3 states "A previous systematic review of sapovirus in African countries revealed that rotavirus..." Not sure why the authors are referring to a sapovirus paper for rotavirus prevalence and the reference used for the study is incorrect - should more likely be 4 or 24 and not 14. Please correct the sentence and the reference.

Is the work clearly and accurately presented and does it cite the current literature?

Partly

If applicable, is the statistical analysis and its interpretation appropriate?

Partly

Are all the source data underlying the results available to ensure full reproducibility?

Yes

Is the study design appropriate and is the work technically sound?

Partly

Are the conclusions drawn adequately supported by the results?

Partly

Are sufficient details of methods and analysis provided to allow replication by others?

No

Reviewer Expertise:

Expertise includes epidemiology of enteric viruses

I confirm that I have read this submission and believe that I have an appropriate level of expertise to confirm that it is of an acceptable scientific standard, however I have significant reservations, as outlined above.

# Response

**Published:** 2021-11-16

**Authors:** Maysaa El Zaki

**Affiliations:** Mansoura University, Egypt

Author Response to the reviewer

 Thanks Sir for your effort for the revision.

- *Discussion, paragraph 1: Please rather use low-income, middle-income or high-income as these have been defined by the World Bank rather than underdeveloped which is not specific or well defined. This correction was noted in the previous review and has not been full corrected.*
- **Response:** Corrected. 
- *Discussion, paragraph 2: Please delete the word hospital. The previous review indicated that either outpatient or hospital should be retained and the other word deleted as it was unclear where the study was conducted.*
- **Response:** Deleted. 
- *Discussion, paragraph 3, line 3 states "A previous systematic review of sapovirus in African countries revealed that rotavirus..." Not sure why the authors are referring to a sapovirus paper for rotavirus prevalence and the reference used for the study is incorrect - should more likely be 4 or 24 and not 14. Please correct the sentence and the reference.*
- **Response:** The reference was corrected.

# Peer review: Reviewer response for version 2

**DOI:** 10.5256/f1000research.56913.r85957

**Published:** 2021-11-02

**Authors:** Marta Diez Valcarce

**Affiliations:** 1 Division of Viral Diseases, Centers for Disease Control and Prevention, Atlanta, GA, USA

I accept the manuscript in this new revised form.

Is the work clearly and accurately presented and does it cite the current literature?

Yes

If applicable, is the statistical analysis and its interpretation appropriate?

Yes

Are all the source data underlying the results available to ensure full reproducibility?

Yes

Is the study design appropriate and is the work technically sound?

Yes

Are the conclusions drawn adequately supported by the results?

Yes

Are sufficient details of methods and analysis provided to allow replication by others?

Yes

Reviewer Expertise:

NA

I confirm that I have read this submission and believe that I have an appropriate level of expertise to confirm that it is of an acceptable scientific standard.

# Peer review: Reviewer response for version 2

**DOI:** 10.5256/f1000research.56913.r93206

**Published:** 2021-09-14

**Authors:** Nicola A. Page

**Affiliations:** 1 Center for Enteric Diseases, National Institute for Communicable Diseases (NICD), Johannesburg, South Africa

The paper describes the EIA screening of rotavirus and RT-PCR detection of sapovirus in 100 children with acute diarrhoea enrolled from outpatient clinics in Mansoura, Egypt between January 2019 and February 2020. Children with bacterial or parasite infections were excluded from the study. Researchers collected data on symptoms, date of presentation and area of residence. While the paper has merit, there are a few major challenges with the paper in the current format:

- When reporting viral epidemiology, authors should include person, place and time. The title of the article is misleading in this sense as it does not indicate either place or time and is rather vague on the persons. The authors should consider changing it to “Molecular detection of sapovirus in children under five years with acute gastroenteritis in Mansoura, Egypt between January 2019 and February 2020”. This would include person, place and time – more appropriate for a study describing sapovirus epidemiology.
- Is the work clearly and accurately presented and does it cite the current literature? Some of the literature cites is not appropriate for the statement/argument being made. Problems with references have been detailed in the report below. 
- Is the study design appropriate and does the work have academic merit? While the work may have merit, the study design is a little confusing as it was not clear when the parasite and bacterial screening was done and when the cases were excluded from the study due to detection of enteric parasites and bacteria. It was also not clear if case were enrolled as outpatients or patients admitted to the Mansoura Hospital. This requires some clarification in the methods section. 
- Are sufficient details of methods and analysis provided to allow replication by others? No, there were not sufficient details provided for the PCR reactions. In addition, one of the primers was incorrectly labelled. Corrections to these sections have been suggested in the detailed report below.
- If applicable, is the statistical analysis and its interpretation appropriate? Mostly. When reporting percentage, it is better to provide the numbers used to calculate the prevalence or percentage – make it clearer for the reader to interpret and evaluate the results.
- Are all the source data underlying the results available to ensure full reproducibility? Yes
- Are the conclusions drawn adequately supported by the results? Not all of the conclusions are supported. Sapovirus is not an emerging virus, it was always present as a cause of gastroenteritis in children. What has changed is our ability to detect it. The authors should always include person, place and time when discussing sapovirus prevalence from this or other studies.

**Page 1:**

- Abstract:
   
  - Background, line 1:  Sapovirus has not recently emerged, it was always present but what has changed is our ability to detect it. Rather say that sapovirus has been shown to be an important viral cause of gastroenteritis in children under two years of age. This statement is supported by both the GEMS and MAL-ED studies that used molecular detection methods for sapovirus. 
  - Background, line 2:  The way that this sentence is written is misleading. There is data to support the circulation of sapovirus in children - see GEMS and MAL-ED which were done in multiple countries in Africa, South East Asia and Latin America. It might be more correct to say that there is limited data on sapovirus in Egypt or in Mansoura, Egypt.
  - Background, line 4: Please indicate place and time and be more specific for persons. i.e. children <5 years and include “…in Mansoura, Egypt from January 2019 to February 2020.”
- Methods, line 7: This section is unclear, consider rewriting as follows:  “The cross-sectional study enrolled a 100 children <5 years who presented with acute gastroenteritis at an outpatient clinic in Mansoura, Egypt between January 2019 and February 2020. Clinical data, demographic data and a stool sample was collected from each child. Stools were screened by microscopy for parasites and culture methods for bacteria and excluded from the study if positive for either. Specimens were also screened for rotavirus by enzyme immune assays (EIA) and sapovirus by reverse transcription PCR.
- Results: Please report prevalence as a percentage followed by the numbers used to calculate the percentage i.e. 10% (10/100). This needs to be corrected throughout the paper.
   
  - Results, line 20: Typically all numbers <10 should be written out. While this is no longer a recommendation by the International Committee of Medical Journal Editors, the authors should consider this for the current article under review.
  - Results, line 21-24: Delete this section from “There was an insignificant…” to the end of the section. Only the main findings should be reported in the abstract so if the results are not significant exclude from the abstract.

**Page 3:**

- Introduction, paragraph 2, line 5: This is very vague – please define good. Rather state that the sensitivity and specificity of most RT-PCR detection assays for sapovirus are above 90%. Look at article from Yan et al., 2003 J Med Virol and Svraka et al., 2009 J Clin Micro.
- Introduction, paragraph 2, line 6: The primers used in this study amplify the capsid and not the VP1-RdRp junction. Please modify this sentence to reflect this fact.
- Introduction, paragraph 3, line 1: This is not an appropriate reference for this statement. Please insert an appropriate and original reference, try looking at the World Health Organization or similar global bodies that conduct these calculations.
- Introduction, paragraph 3, line 8: Prevention of sapovirus will include access to clean water and uncontaminated food but also on good hygiene habits (WHO’s five keys to food safety) and hand washing. Please update sentence to reflect this.
- Methods, paragraph 5, line 5: This is unclear. Were the children admitted to the hospital or were they enrolled from the outpatient clinic? When was a stool specimen taken to exclude parasites and bacteria? Please also indicate if you mean children under five years of age or if you included slightly older children.
- Methods, paragraph 5, line 4: “…Mansoura, Egypt. The study was conducted from January 2019 to February 2020. All children provided a stool specimen which was screened for parasites by direct microscopy and bacterial aetiology by culture. Children positive for an enteric parasite or bacteria were excluded from the study.” 
- Methods, ELISA for rotavirus: This section is unnecessary. It is sufficient to say that the EIA was completed according to the manufacturer’s instructions with EIA plates read at 450nm on a Statfax Chromate 4300 - Please provide manufacturers' details for the Statfax 4300.
- Methods, Extraction of RNA and cDNA amplification: Please supply enough detail so that someone else could replicate the assay if they wanted to – in the current format no detail was provided for primer concentration, buffer or kit used, manufacturer of the RNase inhibitor or where the dNTPs or RT enzyme were manufactured, concentration used etc. Please rewrite this section.

**Page 4:**

- Methods, PCR for sapovirus: Primer name incorrect - SLV5749 forward, please correct. Please also provide the concentration that the primers were used at or the reference of the method used in the lab with enough information to be replicated by the person reading it.
- These primers were also originally designed by Yan, H., F. Yagyu, S. Okitsu, O. Nishio, and H. Ushijima. 2003. Detection of norovirus (GI, GII), sapovirus and astrovirus in fecal samples using reverse transcription single-round multiplex PCR. J. Virol. Methods 114:37-44. Please use the original reference and not Svraka. 
- Results - Please report prevalence as a percentage followed by the numbers used to calculate the percentage i.e. 10% (10/100). This needs to be corrected throughout the paper.
- Discussion, line 1: Data from the GEMS and MAL-ED studies support this statement. Have a look at these studies.
- Discussion, line 2: Using the word developing, underdeveloped or developed is not appropriate to describe a country as it is not well defined i.e. all countries could technically be described as developing. Rather use the word low-income or high-income as this has been defined by the World Bank.
- Discussion, paragraph 2:  This paragraph is a repetition of the results and not a discussion. Please rewrite this section. 
- Consider “The present study including children <5 with diarrhoea in an outpatient/hospital (please indicate the correct setting as it is unclear from the article presented) setting showed that the majority of cases experienced fever, vomiting and abdominal pain; common symptoms of viral gastroenteritis. These patients usually present to outpatient clinics and do not require hospital admission except in rare instance of dehydration. The findings support the results noted in this study.” 
- Discussion, paragraph 3, line 4: “A previous study in Nigeria in 2014-2015 revealed…” Please also indicate if children were under 5 years and if by general population you mean everyone over five years or all ages.
- Discussion, paragraph 3, line 8:  Please provide the time period and be more specific about the place and age of the cases - person, place and time.
- Discussion, paragraph 4, line 6: “A previous systematic review…of sapovirus in African countries…”
- Discussion, paragraph 4, line 9:  Please provide the persons, place and time for the stated sapovirus prevalence?

**Page 5:**

- Discussion, paragraph 2, line 7: “..role of improvement of water and sanitation…”
- Discussion, paragraph 2 line 10: “…improving food safety and access to clean water and improved sanitation services…”
- Discussion, paragraph 3, line 3: Change mandatory.
- Discussion, paragraph 4, line 7: “…circulation of enteric viruses including sapovirus…”
- Conclusions – Please remove the word emergence and frequent as these are not appropriate given the data presented. Also include the place i.e. Mansoura, Egypt.

In the current format this paper requires extensive reworking prior to indexing looking at the comments made above.

Is the work clearly and accurately presented and does it cite the current literature?

Partly

If applicable, is the statistical analysis and its interpretation appropriate?

Partly

Are all the source data underlying the results available to ensure full reproducibility?

Yes

Is the study design appropriate and is the work technically sound?

Partly

Are the conclusions drawn adequately supported by the results?

Partly

Are sufficient details of methods and analysis provided to allow replication by others?

No

Reviewer Expertise:

Expertise includes epidemiology of enteric viruses

I confirm that I have read this submission and believe that I have an appropriate level of expertise to confirm that it is of an acceptable scientific standard, however I have significant reservations, as outlined above.

# Response

**Published:** 2021-09-15

**Authors:** Maysaa El Zaki

**Affiliations:** Mansoura University, Egypt

Thanks for the reviewer comments and for the time to review the article. I have made the required changes

  

- *When reporting viral epidemiology, authors should include person, place and time. The title of the article is misleading in this sense as it does not indicate either place or time and is rather vague on the persons. The authors should consider changing it to “Molecular detection of sapovirus in children under five years with acute gastroenteritis in Mansoura, Egypt between January 2019 and February 2020”.*
- **Response: **Done.
- *Is the work clearly and accurately presented and does it cite the current literature? Some of the literature cites is not appropriate for the statement/argument being made. Problems with references have been detailed in the report below. Is the study design appropriate and does the work have academic merit? While the work may have merit, the study design is a little confusing as it was not clear when the parasite and bacterial screening was done and when the cases were excluded from the study due to detection of enteric parasites and bacteria.When presented with acute gastroenteritis stool samples was taken and examined for parasite and bacterial pathogens and the remaining sample was preserved if negative the sample was included in the study. It was also not clear if case were enrolled as outpatients or patients admitted to the Mansoura Hospital. This requires some clarification in the methods section.*
- **Response: **Outpatients and added.
- *Are sufficient details of methods and analysis provided to allow replication by others? No, there were not sufficient details provided for the PCR reactions. In addition, one of the primers was incorrectly labelled. Corrections to these sections have been suggested in the detailed report below. If applicable, is the statistical analysis and its interpretation appropriate? Mostly. When reporting percentage, it is better to provide the numbers used to calculate the prevalence or percentage – make it clearer for the reader to interpret and evaluate the results.*
- **Response: **Added.
- *Are all the source data underlying the results available to ensure full reproducibility? Yes. Are the conclusions drawn adequately supported by the results? Not all of the conclusions are supported. Sapovirus is not an emerging virus, it was always present as a cause of gastroenteritis in children. What has changed is our ability to detect it. The authors should always include person, place and time when discussing sapovirus prevalence from this or other studies.*
- **Response:** Added.

**Page 1:**

- *Abstract: Background, line 1:  Sapovirus has not recently emerged, it was always present but what has changed is our ability to detect it. Rather say that sapovirus has been shown to be an important viral cause of gastroenteritis in children under two years of age. This statement is supported by both the GEMS and MAL-ED studies that used molecular detection methods for sapovirus. Background, line 2:  The way that this sentence is written is misleading. There is data to support the circulation of sapovirus in children - see GEMS and MAL-ED which were done in multiple countries in Africa, South East Asia and Latin America. It might be more correct to say that there is limited data on sapovirus in Egypt or in Mansoura, Egypt.*
- **Respond: **Added there is limited data on sapovirus in Egypt or in Mansoura, Egypt.
- *Background, line 4: Please indicate place and time and be more specific for persons. i.e. children <5 years and include “…in Mansoura, Egypt from January 2019 to February 2020.”*
- **Response:** Done.
- *Methods, line 7: This section is unclear, consider rewriting as follows:  “The cross-sectional study enrolled a 100 children <5 years who presented with acute gastroenteritis at an outpatient clinic in Mansoura, Egypt between January 2019 and February 2020. Clinical data, demographic data and a stool sample was collected from each child. Stools were screened by microscopy for parasites and culture methods for bacteria and excluded from the study if positive for either. Specimens were also screened for rotavirus by enzyme immune assays (EIA) and sapovirus by reverse transcription PCR.*
- **Response: **Done.
- *Results: Please report prevalence as a percentage followed by the numbers used to calculate the percentage i.e. 10% (10/100). This needs to be corrected throughout the paper. Results, line 20: Typically all numbers <10 should be written out. While this is no longer a recommendation by the International Committee of Medical Journal Editors, the authors should consider this for the current article under review.*
- **Response: **Done.
- *Results, line 21-24: Delete this section from “There was an insignificant…” to the end of the section. Only the main findings should be reported in the abstract so if the results are not significant exclude from the abstract.*
- **Response**: Done.

**Page 3:**

- *Introduction, paragraph 2, line 5: This is very vague – please define good. Rather state that the sensitivity and specificity of most RT-PCR detection assays for sapovirus are above 90%. Look at article from Yan et al., 2003 J Med Virol and Svraka et al., 2009 J Clin Micro*.
- **Response:** Done.
- *Introduction, paragraph 2, line 6: The primers used in this study amplify the capsid and not the VP1-RdRp junction. Please modify this sentence to reflect this fact.*
- **Response: **Done.
- *Introduction, paragraph 3, line 1: This is not an appropriate reference for this statement. Please insert an appropriate and original reference, try looking at the World Health Organization or similar global bodies that conduct these calculations.*
- **Response: **Done.
- *Introduction, paragraph 3, line 8: Prevention of sapovirus will include access to clean water and uncontaminated food but also on good hygiene habits (WHO’s five keys to food safety) and hand washing. Please update sentence to reflect this.*
- **Response: **Added.
- *Methods, paragraph 5, line 5: This is unclear. Were the children admitted to the hospital or were they enrolled from the outpatient clinic? When was a stool specimen taken to exclude parasites and bacteria? Please also indicate if you mean children under five years of age or if you included slightly older children. Methods, paragraph 5, line 4: “…Mansoura, Egypt. The study was conducted from January 2019 to February 2020. All children provided a stool specimen which was screened for parasites by direct microscopy and bacterial aetiology by culture. Children positive for an enteric parasite or bacteria were excluded from the study.” *
- **Response: **Done.

**Page 4:**

- *Methods, PCR for sapovirus: Primer name incorrect - SLV5749 forward, please correct. Please also provide the concentration that the primers were used at or the reference of the method used in the lab with enough information to be replicated by the person reading it.These primers were also originally designed by Yan, H., F. Yagyu, S. Okitsu, O. Nishio, and H. Ushijima. 2003. Detection of norovirus (GI, GII), sapovirus and astrovirus in fecal samples using reverse transcription single-round multiplex PCR. J. Virol. Methods 114:37-44. Please use the original reference and not Svraka. Results - Please report prevalence as a percentage followed by the numbers used to calculate the percentage i.e. 10% (10/100). This needs to be corrected throughout the paper. Discussion, line 1: Data from the GEMS and MAL-ED studies support this statement. Have a look at these studies.*
- ** Response: **Added.
- *Discussion, line 2: Using the word developing, underdeveloped or developed is not appropriate to describe a country as it is not well defined i.e. all countries could technically be described as developing. Rather use the word low-income or high-income as this has been defined by the World Bank.*
- **Response:** Done.
- *Discussion, paragraph 2:  This paragraph is a repetition of the results and not a discussion. Please rewrite this section. *
- **Response:** Done.
- *Consider “The present study including children <5 with diarrhoea in an outpatient/hospital (please indicate the correct setting as it is unclear from the article presented) setting showed that the majority of cases experienced fever, vomiting and abdominal pain; common symptoms of viral gastroenteritis. These patients usually present to outpatient clinics and do not require hospital admission except in rare instance of dehydration. The findings support the results noted in this study.”* 
- **Response: **Done.
- *Discussion, paragraph 3, line 4: “A previous study in Nigeria in 2014-2015 revealed…” Please also indicate if children were under 5 years and if by general population you mean everyone over five years or all ages.*
- **Response: **Below five, added.
- *Discussion, paragraph 3, line 8:  Please provide the time period and be more specific about the place and age of the cases - person, place and time.*
- **Response: **Done.
- *Discussion, paragraph 4, line 6: “A previous systematic review…of sapovirus in African countries…”*
- **Response** ***:** *Done.
- *Discussion, paragraph 4, line 9:  Please provide the persons, place and time for the stated sapovirus prevalence?*
- **Response:** Done. 

**Page 5:**

- *Discussion, paragraph 2, line 7: “..role of improvement of water and sanitation…” Discussion, paragraph 2 line 10: “…improving food safety and access to clean water and improved sanitation services…”*
- **Response: **Done.
- *Discussion, paragraph 3, line 3: Change mandatory.*
- **Response:** Done. 
- *Discussion, paragraph 4, line 7: “…circulation of enteric viruses including sapovirus…”*
- **Response: **Done.
- *Conclusions – Please remove the word emergence and frequent as these are not appropriate given the data presented. Also include the place i.e. Mansoura, Egypt.*
- **Response:** Done.

# Peer review: Reviewer response for version 1

**DOI:** 10.5256/f1000research.33041.r83889

**Published:** 2021-05-12

**Authors:** Marta Diez Valcarce

**Affiliations:** 1 Division of Viral Diseases, Centers for Disease Control and Prevention, Atlanta, GA, USA

The article titled 'Molecular study of sapovirus in acute gastroenteritis in children: a cross-sectional study' by El Sayed Zaki *et al*. includes some very interesting data on the prevalence of sapovirus in Egypt.

 Overall, the article is well presented but there are a few minor comments that I would like to make, and also some edits that I consider necessary for clarity and accuracy.

- In the abstract section, results paragraph, I think it could be informative which months are considered winter season in Egypt, as this varies in different parts of the world.
- In the introduction I would suggest to write the family *Caliciviridae* in italics. Also, and that is a major edit that need to be done, the sapovirus genus contains or can be divided into 15 genogroups, not genotypes.
- I am not sure what the authors mean when they say that the primers used depend upon the use of a segment from VP1 encoding gene compared to the RdRp region. I am not aware of any comparison made between the capsid and the polymerase for classification of sapovirus. In some cases, both regions are sequenced to give a more accurate classification, but not compared. Maybe the authors could rewrite this sentences so they are clearer.
- The third paragraph of the introduction mentioned a reference but I think the authors misinterpreted the findings of that work. What the article said is that globally, diarrhea is the second cause of mortality and the most common cause of illness in children younger than 5, and that it causes approximately 2 million deaths per year. But that is diarrhea, from any cause, not only sapovirus. Please correct.
- In the methods section, under the stool samples paragraph the acronym for the molecular detection method used is incorrect, it should read RT-PCR and not RT-PVR.
- Also in the methods section, when the mentioned the Ridascreen method to detect rotavirus, I think they should refer to the method as a semiquantitative one, since as they mentioned, the color intensity is proportional to the concentration of rotavirus present compared with the control.
- I also suggest to rewrite the part in which they explain how the kit uses monoclonal antibodies that specifically react with the VP6 protein (not the protein of the six viral genes, please correct).
- In the statistical analysis paragraph, if results were expressed as numbers and percentages, those are quantitative values, not qualitative. Please correct.
- In the discussion section, the fifth paragraph, I suggest to replace the word 'management' for 'treatment'. Also, I would rewrite the sentence and instead of using the word 'argument', I would say something like: There are conflicting/contradictory opinions about the role of water sanitation improvement in the prevention of sapovirus …
- I suggest to replace the expression 'an insignificant difference' for 'no significant difference', throughout the manuscript. Similarly, I also suggest to just mention acute gastroenteritis with all the letters once, with the acronym AGE between brackets, and from then on just use AGE throughout the article.  

Is the work clearly and accurately presented and does it cite the current literature?

Yes

If applicable, is the statistical analysis and its interpretation appropriate?

Yes

Are all the source data underlying the results available to ensure full reproducibility?

Yes

Is the study design appropriate and is the work technically sound?

Yes

Are the conclusions drawn adequately supported by the results?

Yes

Are sufficient details of methods and analysis provided to allow replication by others?

Yes

Reviewer Expertise:

Molecular epidemiology of calicivurs

I confirm that I have read this submission and believe that I have an appropriate level of expertise to confirm that it is of an acceptable scientific standard, however I have significant reservations, as outlined above.

# Response

**Published:** 2021-05-15

**Authors:** Maysaa El Zaki

**Affiliations:** Mansoura University, Egypt

Dear Dr. Diez Valcarce, 

 Thanks for your valuable comments. I have made all the required corrections.

 Best Regards.
